# Contemporary management of the open abdomen: standards, challenges, and future directions

**DOI:** 10.1007/s00423-026-04080-9

**Published:** 2026-05-12

**Authors:** Miloš Kňazovický, Ivan Kováč, Barbora Romžová, Róbert Šimon, Jana Kaťuchová

**Affiliations:** 1 1st Department of Surgery, Louise Pasteur University Hospital and Pavol Jozef Šafárik University, Rastislavova 43, Košice, 04190, Slovak Republic; 2 2nd Department of Surgery, Louise Pasteur University Hospital and Pavol Jozef Šafárik University, Rastislavova 43, Košice, 04190 Slovak Republic; 3 Department of Plastic, Reconstructive, and Aesthetic Surgery, Louise Pasteur University Hospital, Košice, Slovak Republic

**Keywords:** Open abdomen, Damage control surgery, Temporary abdominal closure, Fascial closure, Enteroatmospheric fistula

## Abstract

**Objective:**

The open abdomen (OA) is a key strategy in damage control surgery (DCS) for severe intra-abdominal pathology but is associated with considerable morbidity, including enteroatmospheric fistula (EAF) and failure of fascial closure. This narrative review summarizes current evidence on indications, temporary abdominal closure (TAC), timing of closure, and supportive care.

**Methods:**

A structured PubMed/MEDLINE, Embase, Scopus, and Web of Science search was performed up to January 2025 in accordance with PRISMA principles. Relevant systematic reviews, meta-analyses, clinical guidelines, registry analyses, and prospective studies published in English were included. Given the narrative design, no meta-analysis was conducted.

**Results:**

Negative pressure wound therapy (NPWT) combined with dynamic fascial traction represents a preferred TAC strategy, achieving primary closure rates up to 80–90% in selected patients. Early fascial closure (within 5–7 days) is associated with fewer complications, particularly EAF. Early enteral nutrition (24–48 h), when feasible, may support fascial approximation. However, available evidence is largely observational and heterogeneous, with substantial inter-institutional variability.

**Conclusion:**

Optimal OA management requires appropriate indication, standardized TAC strategies, and a proactive approach to early closure. When primary closure is not feasible, a planned ventral hernia remains a safe alternative. High-quality prospective studies are needed to strengthen the evidence base.

## Introduction

The management of laparotomy wounds using the open abdomen (OA) technique represents a life-saving strategy of last resort in carefully selected clinical scenarios. The concept was first described by William Heneage Ogilvie in the context of war-related abdominal injuries [1]. Since its initial description, the OA technique has undergone substantial evolution and has remained the subject of ongoing debate. In contemporary practice, however, it is widely accepted as a valid therapeutic approach when applied according to well-defined indications.

In 2018, the World Society of Emergency Surgery (WSES) published recommendations for the management of critically ill patients undergoing damage control surgery (DCS) [2]. Both traumatic and non-traumatic patients with severe intra-abdominal pathology are at high risk of developing septic complications, intra-abdominal hypertension (IAH), and abdominal compartment syndrome (ACS), which may ultimately progress to multiple organ failure (MOF). In this setting, the OA approach may represent the only viable option, particularly in the presence of severe visceral edema preventing primary fascial closure, while also allowing effective source control and facilitating planned re-explorations.

The implementation of DCS and OA strategies has been associated with improved survival in a highly selected population of critically ill patients with hemodynamic instability due to trauma, abdominal sepsis, severe acute pancreatitis, or major vascular events. Although overall mortality in this group remains high (approximately 19–41%), these approaches are considered to improve survival relative to otherwise expected fatal outcomes [3, 4].

A common underlying pathophysiological mechanism is that aggressive fluid resuscitation can cause capillary leakage leading to visceral edema and increased intra-abdominal pressure [5, 6]. Goal-directed fluid therapy may consequently contribute to a reduction in IAH; therefore, a restrictive fluid strategy should be considered where clinically feasible [7].

However, despite its life-saving potential, OA is associated with considerable morbidity, including enteroatmospheric fistula (EAF), loss of abdominal domain, complex ventral hernia formation, prolonged intensive care unit (ICU) course, frozen abdomen, infectious complications, and significant fluid and protein losses [8].

Despite advances in temporary abdominal closure (TAC) techniques and perioperative critical care, achieving early fascial closure while minimizing complications remains a major clinical challenge. Moreover, the available evidence is largely heterogeneous and predominantly observational. Therefore, this narrative review aims to critically appraise current evidence on the indications, management strategies, and outcomes associated with the OA technique in patients undergoing DCS.

## Materials and methods

A narrative literature review was conducted in accordance with PRISMA (Preferred Reporting Items for Systematic Reviews and Meta-Analyses) principles for transparent reporting. A comprehensive search of PubMed, Embase, Scopus, and Web of Science was performed to identify relevant studies published up to January 2025, without restrictions on publication date.

The search strategy combined the following keywords and their variations using Boolean operators: “open abdomen,” “open abdomen technique,” “laparostomy,” “damage control surgery,” “open abdomen classification,” “open abdomen closure,” “temporary abdominal closure,” “definitive abdominal closure,” “indications for open abdomen,” “surgical management of open abdomen,” “nutritional support,” “intra-abdominal hypertension,” “abdominal compartment syndrome,” and “enteroatmospheric fistula.”

Titles and abstracts were screened for relevance, followed by full-text assessment of eligible articles. Studies were selected based on their relevance to the clinical management of patients undergoing the open abdomen technique. Given the narrative design of the review, no formal quality assessment or meta-analysis was performed.

The objective of this review was to synthesize current evidence and provide practical recommendations for the management of patients undergoing the open abdomen technique.

## Results

### Intra-abdominal hypertension and abdominal compartment syndrome: definitions and classification

Intra-Abdominal hypertension (IAH) and abdominal compartment syndrome (ACS) are key conditions in patients requiring OA management. According to the updated World Society of the Abdominal Compartment Syndrome (WSACS) guidelines, intra-abdominal pressure (IAP) is typically measured via the bladder [9].

IAH is defined as a sustained IAP ≥ 12 mmHg and is graded as follows: Grade I (12–15 mmHg), Grade II (16–20 mmHg), Grade III (21–25 mmHg), and Grade IV (> 25 mmHg). ACS is defined as a sustained IAP > 20 mmHg associated with new organ dysfunction.

ACS is further classified as primary (intra-abdominal origin), secondary (extra-abdominal origin), or recurrent. Early recognition of IAH/ACS is essential for timely intervention, including decompressive laparotomy and OA management [9].

## Classification of the open abdomen

The increasing incidence of IAH, which may frequently progress to ACS, has necessitated the formulation of internationally accepted definitions, management strategies, and a standardized classification system to guide clinical practice. The need for an OA classification system arose from several considerations, including the standardization of clinical status, the harmonization of therapeutic recommendations for OA management, and the ability to compare outcomes among specialized research groups investigating this condition [10].

As a result of these efforts, the first comprehensive classification system for patients managed with the OA technique was introduced in 2009. Subsequently, in 2016, the original classification was revised and updated by a group of authors led by Michael Björck, resulting in a modified classification system that is currently widely adopted in clinical practice (Table 1) [11].

**Table 1:** Revised classification of the open abdomen (OA) technique.*Source: adapted from Michael Björck et al. [4].*

Grade	Description
**1 A**	Clean open abdomen, without fixation or adhesions
**1B**	Contaminated open abdomen, without fixation or adhesions
**1 C**	Open abdomen with enteric leak, without fixation
**2 A**	Clean open abdomen with developing fixation
**2B**	Contaminated open abdomen with developing fixation
**2 C**	Open abdomen with enteric leak and developing fixation
**3 A**	Clean *frozen abdomen*
**3B**	Contaminated *frozen abdomen*
**4**	*Frozen abdomen* with entero-atmospheric fistula

The classification proposed by Michael Björck et al. reflects the progressive severity of the open abdomen condition. Lower grades (1 A–1 C) represent early stages without significant fixation of the abdominal wall or viscera, whereas grades 2 A–2 C indicate the development of adhesions and fixation, which may complicate delayed fascial closure. Advanced stages (3 A–3B) correspond to the so-called *frozen abdomen*, characterized by dense adhesions and loss of abdominal domain, while grade 4 represents the most severe form, defined by the presence of an entero-atmospheric fistula

## Indications for the open abdomen technique

Indications for the OA technique can be broadly categorized as physiological, anatomical, and logistical. Physiological indications include situations such as hemodynamic instability requiring emergent laparotomy within the framework of DCS. Anatomical indications arise when primary abdominal closure is not feasible due to loss of abdominal wall soft tissue, most commonly in the setting of trauma or severe intra-abdominal sepsis, or when there is a high risk of developing ACS [12].

Logistical indications refer to clinical situations in which a planned re-exploration (*second-look laparotomy*) is anticipated. In general, the indications for the OA technique can be divided into two principal groups: those related to traumatic and those related to non-traumatic patients (Table 2) [2]. The relative frequency of these indications exhibits significant regional variation: in North American cohorts, peritonitis (38.7%) and trauma - gunshot and stab wounds − (34.7%) had a similar distribution, while in the European and the Asiatic continent prevailed peritonitis [13].

**Table 2:** Indications for the open abdomen (OA) technique in traumatic and non-traumatic patients *Source: adapted form Frederico Cocccolini et al.* [2]. *IAP – intra-abdominal pressure; IAH – intra-abdominal hypertension; ACS – Abdominal Compartment Syndrome.*

Patient group	Indications
**Trauma**	• Severe abdominal trauma associated with the “lethal triad” of metabolic acidosis (pH < 7.2), hypothermia (T < 34 °C), and coagulopathy, often accompanied by persistent hypotension
	• Presence of significant risk factors for the development of intra-abdominal hypertension (IAH) or ACS
	• Ongoing intra-abdominal contamination or infection without the possibility of definitive source control
	• Suspected or confirmed intestinal hypoperfusion following abdominal injury
**Non-trauma**	• Intra-abdominal pressure (IAP) > 20 mmHg associated with organ dysfunction when conservative management fails
	• Persistent intra-abdominal infection without the possibility of definitive source control
	• High risk of ACS in patients with severe acute pancreatitis
	• High risk of ACS in patients with rupture of Abdominal Aortic Aneurysm or acute mesenteric ischemia

The indications for the OA technique differ between traumatic and non-traumatic conditions, reflecting differences in underlying pathophysiology and clinical priorities. In trauma patients, the OA approach is most commonly employed as part of damage control surgery in the setting of physiological exhaustion and the presence of the “lethal triad.” In contrast, in non-traumatic patients the technique is more frequently used to manage severe intra-abdominal sepsis, abdominal compartment syndrome, or conditions associated with significant visceral edema and impaired abdominal wall closure. In both groups, timely recognition of these indications is essential to prevent progression to organ failure and to optimize patient outcomes

Furthermore, the WSACS reports that while ACS complicates roughly 5–15% of major trauma cases, its incidence may reach 30% in non-traumatic populations requiring massive fluid resuscitation for septic shock [9]. This regional refinement is clinically significant, as non-traumatic patients typically demonstrate lower primary fascial closure rates and increased morbidity compared to their traumatic counterparts.

## Surgical management and choice of operative approach

The primary goal of surgical management using the OA technique is to achieve definitive fascial and abdominal wall closure. This objective must be pursued while simultaneously addressing the risk of developing ACS and septic complications, the most significant of which is the formation of an EAF [9, 14].

**Fig. 1:**
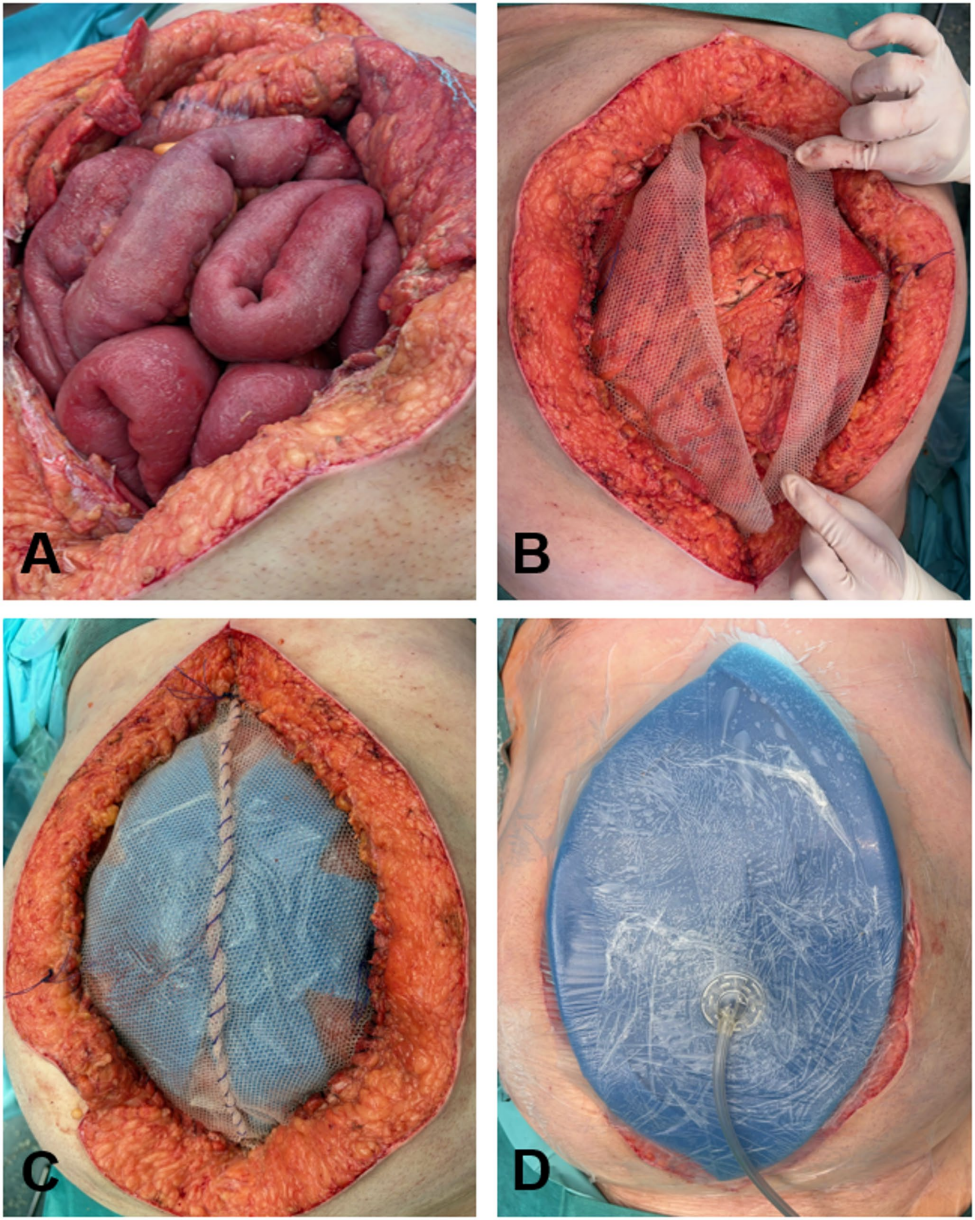
Open abdomen management after midline laparotomy. (**A**) Bowel wall edema. (**B**) Polypropylene mesh sutured to the fascial edges. (**C**) Midline approximation of the mesh. (**D**) Application of negative pressure wound therapy (NPWT) Source First Department of Surgery, Pavol Jozef Šafárik University, Faculty of Medicine and Louis Pasteur University Hospital, Košice, Slovakia

In emergency surgery, midline laparotomy (a long vertical incision along the linea alba) remains the most commonly used surgical approach, primarily due to its rapid execution and technical simplicity (Fig. 1). In cases of established ACS, decompressive laparotomy has demonstrated high effectiveness in reducing IAP [15]. However, laparotomy in the setting of an OA is associated with a significant risk of EAF formation, reported in an incidence of approximately 5–25% of patients depending on underlying pathology and management strategy, as well as a relatively frequent failure to achieve definitive fascial closure (DFC) [8, 16]. These limitations have stimulated the search for alternative operative approaches.

**Fig. 2:**
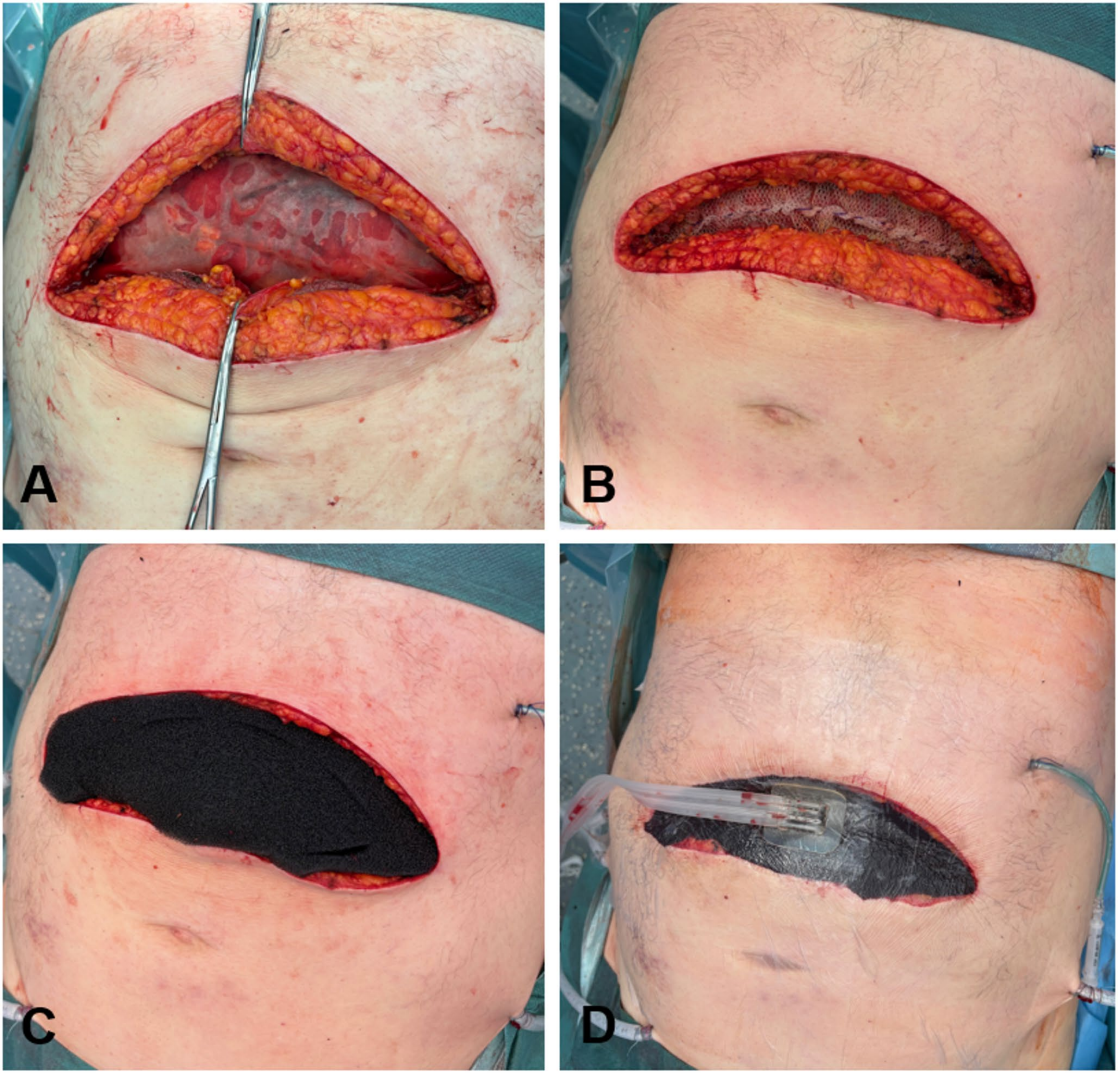
Open abdomen management after transverse laparotomy. (**A**) Anti-adhesion semi-permeable membrane. (**B**) Polypropylene mesh sutured to the fascial edges providing dynamic traction. (**C**) Polyurethane foam tailored to wound dimensions. (**D**) Application of negative pressure wound therapy (NPWT) Source First Department of Surgery, Pavol Jozef Šafárik University, Faculty of Medicine and Louis Pasteur University Hospital, Košice, Slovakia

One promising alternative is transverse laparotomy, the effectiveness of which has been reported in several recent studies (Fig. 2). The procedure requires the same surgical instrumentation and can be performed with only slightly longer operative time compared with midline laparotomy. Importantly, the general principles of OA management remain identical regardless of whether a midline or transverse incision is used.

In emergency surgical practice, transverse laparotomy in the setting of OA has shown particular benefit in the treatment of severe acute pancreatitis [17]. Increasing evidence also suggests that a transverse abdominal incision may provide improved rates of abdominal closure in patients with septic peritonitis. In both clinical conditions, however, delayed fascial closure remains a major challenge. The lower rate of fascial dehiscence and complications associated with transverse laparotomy is thought to result from the thicker tissue coverage and improved vascularization of the lateral abdominal wall [18]. Moreover, the physiological orientation of the incision preserves the integrity of the rectus abdominis muscles while leaving the linea alba intact. This significantly reduces fascial retraction, which represents the primary obstacle to successful delayed fascial closure. Nevertheless, midline laparotomy continues to offer advantages in terms of shorter operative times and easier extension of the incision when wider abdominal exposure is required [15].

A wide spectrum of TAC techniques is currently used in clinical practice. Among these, methods utilizing NPWT are generally considered the most effective. In contrast, passive techniques without negative pressure, such as the *Bogotá bag*, should be reserved mainly for situations with limited material resources [2, 19]. A specific technique historically used in OA management is skin-only closure, in which the skin is closed while the fascia remains open. This method was removed from official recommendations by the WSES in 2018 due to several disadvantages, including the inability to control intra-abdominal fluid drainage, interference with radiological imaging, and a high incidence of ACS, evisceration, and skin-related complications. Despite these concerns, the technique has recently regained some attention, particularly in the context of military surgery and extreme emergency scenarios. Skin-only closure is extremely rapid, inexpensive, and widely available, allowing rapid reduction of heat and fluid loss. In critically ill hemodynamically unstable patients, the procedure can therefore be completed within a very short time interval, which represents its main advantage when rapid termination of life-saving laparotomy is required [20].

In the management of OA, the surgeon’s primary effort should focus on achieving early DFC while preventing the development of EAF. A key strategy to prevent progression toward the so-called *frozen abdomen* is the reduction of adhesion formation between intestinal loops and the abdominal wall, together with prevention of fascial retraction. These objectives are effectively addressed by NPWT combined with dynamic fascial traction using a mesh, a technique first described in 2007 [21]. The principle of this method involves placement of a specialized anti-adhesive visceral protective layer that separates the abdominal viscera from the abdominal wall. This interface not only mechanically prevents adhesion formation but is also essential for maintaining bowel wall integrity and mitigating the risk of EAF formation [3, 22]. At the same time, a polypropylene mesh is sutured to the fascial edges to counteract the lateral traction forces of the abdominal wall musculature and prevent progressive fascial retraction.

The main advantages of polypropylene mesh traction include its high mechanical resistance to traction forces, the possibility of repeated abdominal re-exploration, and the gradual approximation of fascial edges during subsequent surgical revisions. Unlike absorbable materials such as Vicryl mesh, which provide only temporary stabilization and are prone to loss of tensile strength in the presence of enzymatic degradation, the non-absorbable nature of polypropylene allows for sustained tension [21].

The combination of NPWT with dynamic fascial traction has demonstrated the highest success rates in achieving delayed fascial and is currently recommended as the preferred TAC technique, even in cases of septic peritonitis or severe intra-abdominal contamination [2]. A refined and widely adopted iteration of this approach is Vacuum-Assisted Wound Closure and Mesh-Mediated Fascial Traction (VAWCM).

The VAWCM technique integrates the benefits of negative pressure with a mechanical mechanical tension system. In this procedure, a temporary prosthetic mesh (typically polypropylene) is sutured to the fascial edges and used as a bridge. The NPWT system is then applied over the mesh. During subsequent surgical re-explorations, the mesh is incised in the midline, the abdomen is washed, and the mesh is re-tightened by suturing it back together, effectively pulling the fascial edges toward the midline. Clinical data indicate that this combined approach achieves DFC rates ranging from 77% to 100% [23, 24]. It should be emphasized that NPWT used alone, without the addition of a dynamic traction component, is associated with significantly lower closure rates—often reported between 18% and 59% and a prolonged interval to achieving definitive closure. This disparity is primarily attributed to the inability of standalone NPWT to counteract the lateral retraction of the fascial edges [25, 26]. VAWCM effectively mitigates these risks by providing constant, adjustable medial tension while simultaneously managing peritoneal exudate and reducing visceral edema via negative pressure.

### Strategy and timing of temporary abdominal closure

Successful management of the OA is time-dependent and requires early transition from decompression to active fascial traction. Dynamic fascial traction should be initiated during the index operation or, at the latest, within the first 24–48 h. In hemodynamically stabilized patients, early traction—using either mesh-mediated (horizontal) or vertical systems—should be applied promptly to prevent lateral fascial retraction. Delayed initiation (> 48 h) is associated with increased risk of fascial fixation, loss of abdominal domain, and development of a frozen abdomen [27].

Management should follow a structured, stepwise approach. After initial decompression and source control, with adequate visceral protection, early fascial traction must be established. This strategy utilizes the residual elasticity of the abdominal wall before the onset of inflammation-related fibrosis. Techniques such as VAWCM or vertical traction systems (e.g., Fasciotens^®^) are recommended to maintain fascial approximation and preserve the potential for definitive closure [28, 29].

Planned re-explorations should be performed at 24–48-hour intervals, with progressive adjustment of traction as visceral edema decreases. Continuous, controlled tension facilitates fascial medialization through stress relaxation and tissue creep, thereby preserving abdominal domain and reducing the need for complex reconstructive procedures.

Definitive fascial closure should be achieved as early as clinically feasible, ideally within 5–7 days [30]. Prolonged OA duration is associated with increased risk of EAF formation and infectious complications. Early, dynamic fascial traction is therefore a key component of contemporary OA management aimed at minimizing morbidity [2].

## Nutritional support

In the contemporary management of patients treated with the OA technique, increasing emphasis is placed on the benefits of early enteral nutrition (EN). However, it must be emphasized that the current evidence base remains limited and is largely derived from older literature. Furthermore, many existing studies address critically ill ICU patients in general rather than specifically those with an open abdomen; nevertheless, these findings appear largely transferable to the OA population when interpreted alongside the ESPEN guidelines on clinical nutrition in the ICU and surgery [31]. Notably, in critically ill patients with OA/NPWT, the use of enteral feeding within 7 days after surgery has been associated with better clinical outcomes [32].

According to current recommendations, enteral feeding should be initiated as soon as hemodynamic stability has been achieved, and adequate visceral perfusion has been ensured. A fundamental prerequisite is the presence of a viable and functional gastrointestinal tract, while a residual small bowel length of less than 75 cm is generally considered a relative contraindication to enteral feeding [33].

Whenever feasible, placement of a nasojejunal feeding tube during the initial surgical procedure is recommended. If insertion is not possible due to significant edema or obstruction of the duodenum, the creation of a feeding jejunostomy may represent an appropriate alternative.

Initiation of EN within the first 24–48 h has been shown to significantly improve wound healing, accelerate the achievement of DFC, and mitigate the hypercatabolic state while preserving the integrity of the intestinal mucosal barrier [34, 35]. In addition, early enteral feeding reduces the incidence of nosocomial pneumonia, decreases the risk of developing EAF, and ultimately shortens the length of hospital stay, thereby reducing overall treatment costs [35, 36].

Compared with total parenteral nutrition (TPN), early EN significantly lowers the risk of septic complications, which is particularly important in patients with abdominal trauma or concomitant traumatic brain injury [37]. Prolonged TPN has been associated with intestinal mucosal atrophy, increased bacterial translocation, and progressive intestinal wall edema, all of which may increase the risk of EAF formation [38]. For this reason, TPN should generally be reserved for selected situations such as intestinal malabsorption or the presence of a high-output intestinal fistula.

Recent evidence also suggests that EN in patients with OA may contribute to improved lymphatic flow, enhanced venous return, and reduction of visceral edema. Clinical outcomes strongly support this approach, as patients receiving early enteral feeding achieve rates of primary abdominal wall closure of up to 74%, compared with approximately 49% in patients in whom nutritional support is initiated later [35].

## Definitive closure of the abdominal wall

In cases of prolonged OA management where primary fascial closure cannot be achieved due to significant fascial retraction, a strategy of planned ventral hernia is often adopted. In patients with persistent visceral edema, TAC devices are typically removed after approximately 2–3 weeks. The primary objective during this phase is protection of the intra-abdominal organs, achieved either through skin closure or by promoting granulation tissue formation followed by coverage with split-thickness skin grafts. Definitive abdominal wall reconstruction is performed in a delayed manner, usually 6–12 months later, when inflammatory adhesions have subsided and tissue conditions have stabilized [39].

**Fig. 3:**
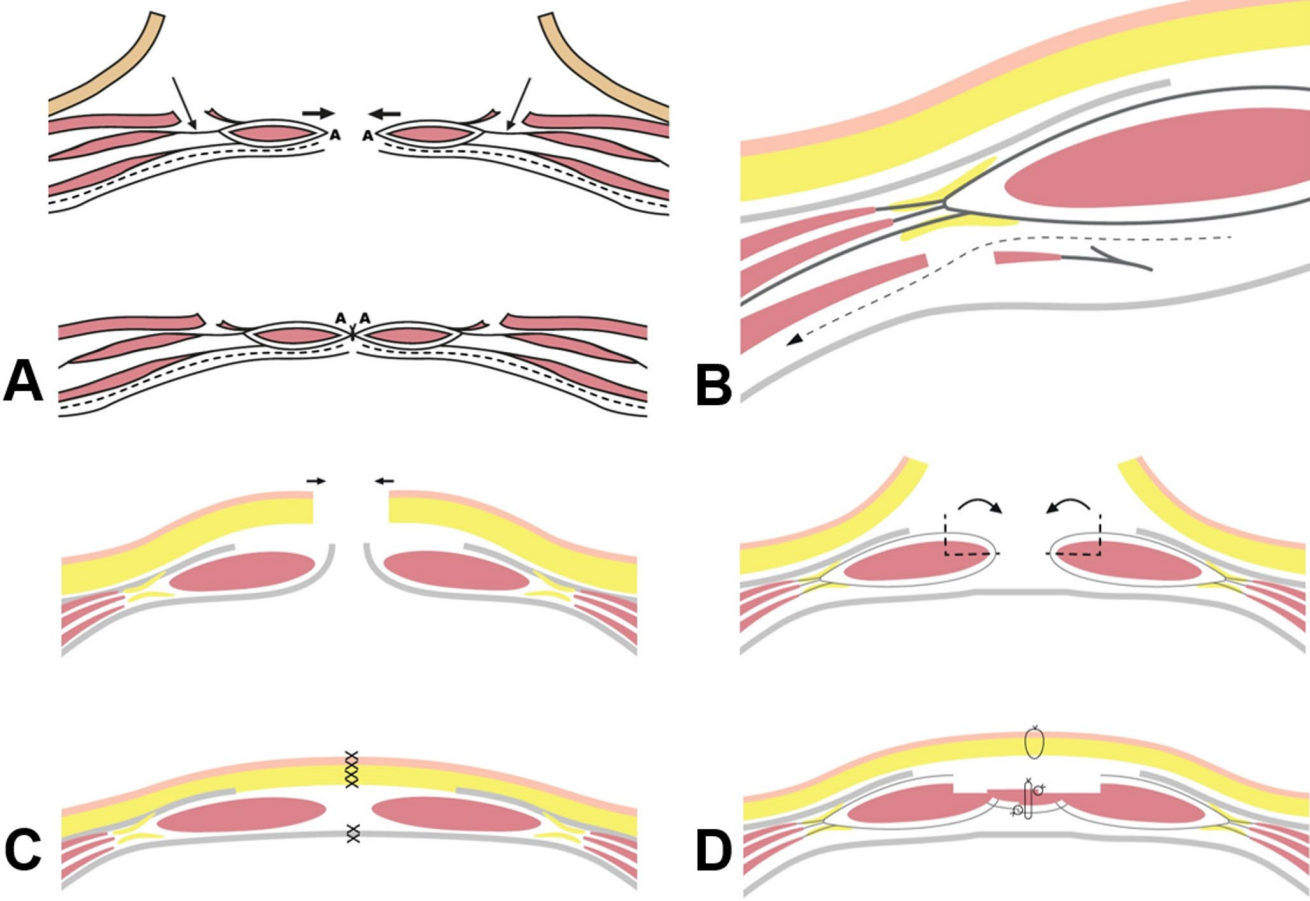
Component separation techniques. (**A)** Anterior component separation (ACS). (**B**) Posterior component separation (PCS). (**C**) Modified component separation with turnover flap of the anterior rectus sheath (RSTF). (**D**) Myofascial rectus abdominis splitting flap **Source** First Department of Surgery, Pavol Jozef Šafárik University, Faculty of Medicine and Louis Pasteur University Hospital, Košice, Slovakia

Surgical repair techniques are generally selected according to the condition of the skin envelope. When adequate skin coverage and mobility are preserved, reconstruction may be achieved through bridging mesh repair or the use of the component separation technique (CST) (Fig. 3). The fundamental principle of CST involves mobilization of the musculo-fascial components of the abdominal wall, particularly the rectus abdominis complex, allowing restoration of the midline. Component separation can be performed via anterior or posterior approaches, including the transversus abdominis release (TAR) technique, and these procedures are frequently combined with prosthetic mesh reinforcement [40].

The choice of mesh material plays a critical role regarding the risk of infection and hernia recurrence. Synthetic meshes are generally preferred due to their superior mechanical strength and cost-effectiveness. Current evidence suggests that mesh porosity and weight are key determinants of successful tissue integration, with macroporous, medium-weight meshes demonstrating the most favorable balance between durability and resistance to infection [41].

In contaminated surgical fields, synthetic absorbable meshes such as Vicryl or Dexon were historically proposed. However, their rapid degradation within approximately 1–3 months has been associated with unacceptably high recurrence rates. To overcome these limitations, biosynthetic meshes such as GORE BIO-A and Phasix have been developed. These materials degrade more slowly, typically over six months or longer—providing prolonged structural support while potentially reducing infection risk and postoperative pain [42].

Biologic meshes, initially introduced with the expectation of improved performance in contaminated environments, have produced inconsistent results when compared with synthetic materials. Studies, including those by Johannes Atema and colleagues, report significantly higher hernia recurrence rates with biologic meshes, reaching approximately 30%, compared with roughly 7% for synthetic meshes [43].

In cases involving extensive abdominal wall defects with unstable or absent skin coverage, advanced reconstructive techniques may be required. In specialized centers, microvascular free flap reconstruction using the tensor fasciae latae muscle (TFL) has emerged as a valuable option. However, this procedure requires close multidisciplinary collaboration between abdominal and plastic surgeons and is generally reserved for complex cases managed in highly specialized surgical centers [44].

## Discussion

The management of the OA has undergone a substantial transformation over the past decade, evolving from a technique of “last resort” to a highly standardized component of modern emergency surgery. A key historical milestone was the establishment of definitions for IAH and ACS by the WSES in 2013, building upon earlier consensus statements of the Abdominal Compartment Society [9]. These standardized terminological frameworks subsequently enabled the development of evidence-based international recommendations published in 2018, which today form the cornerstone of management algorithms for both traumatic and non-traumatic critically ill patients [2].

Historically, much of the evidence supporting the effectiveness of OA management was derived from relatively small and heterogeneous patient cohorts with inconsistent methodological quality. This limitation created a clear need for a robust international data source. In response, the WSES, in collaboration with the Panamerican Trauma Society, launched the International Register of Open Abdomen (IROA) in 2015. This prospective cohort registry collects data on patient demographics, comorbidities, TAC techniques, and OA-specific complications. Data derived from this large-scale collaborative project suggest that, when utilized within appropriate clinical indications, the OA technique is associated with favorable survival trends in patients undergoing DCS. Preliminary analyses indicate that the observed mortality rate in the IROA cohort is approximately 29.7%, which is notably lower than the rates traditionally anticipated for such critically ill populations [45]. However, it is essential to emphasize that these findings represent a clinical association rather than a direct causal reduction in mortality. The reported effectiveness of the OA strategy is significantly influenced by patient selection and the underlying etiology, with traumatic indications generally demonstrating lower mortality compared to non-traumatic, septic cases.

However, geographical analysis of IROA data published in 2023 revealed substantial differences in survival outcomes between continents. While North American and European centers reported comparable treatment durations and relatively lower mortality rates, outcomes in several Asian regions were notably poorer, with mortality exceeding 40%. These discrepancies reflect not only differences in surgical technique but also variations in health-care infrastructure, intensive care availability, and regional differences in indication criteria for OA [13].

Beyond geographic variability, registry analyses have yielded critical insights into high-risk patient subgroups, particularly those with obesity. Current evidence demonstrates that while OA management in obese patients (BMI ≥ 30 kg/m^2^) does not independently increase mortality, it is associated with a significantly more protracted and complex clinical course. Obesity appears to play a detrimental role in extending the duration of OA treatment, with a direct linear correlation observed between increasing BMI and the length of the open phase. Specifically, obese patients require a significantly longer duration of open treatment compared to non-obese cohorts (9.1 ± 11.5 days vs. 6.3 ± 7.5 days) and experience notably longer ICU stays (21.2 ± 35.2 days vs. 14.2 ± 15.6 days). This augmented treatment duration is intrinsically linked to poorer secondary outcomes. Obese patients exhibit significantly lower rates of primary fascial closure (75.5% vs. 89.5%) and skin closure (79.6% vs. 89.5%) [46]. These findings suggest that the mechanical challenges and the pro-inflammatory environment inherent to obesity contribute to impaired wound healing and accelerated fascial retraction, ultimately hindering definitive abdominal closure.

Parallel to the development of IROA, the European Hernia Society established the EuraHS registry, which includes a dedicated module for OA management. Data from this European database, comprising more than 1,000 patients, provides detailed insight into contemporary practice: https://europeanherniasociety.eu/ehs-open-abdominal-registry. A study published in 2023 reported that with a mean OA duration of 20.8 days, overall mortality reached 27.9%, while the incidence of EAF was approximately 7%. These findings highlight the considerable risks associated with prolonged OA management and emphasize the need for optimized closure strategies [22].

With regard to temporary abdominal closure, contemporary practice has shifted from passive techniques, such as the Bogotá bag, toward active NPWT systems. Historical passive methods were primarily designed for visceral protection and decompression but did not provide active fascial traction, resulting in low rates of DFC, typically below 20–30%, and frequently necessitating a planned ventral hernia strategy due to irreversible fascial retraction [8, 10, 47, 48].

Regarding the choice of surgical approach, midline laparotomy remains the dominant technique in emergency surgery because of its rapid execution and excellent intra-abdominal exposure. Nevertheless, the present review highlights the growing clinical potential of transverse laparotomy, particularly in patients with severe acute pancreatitis. Its anatomical advantages—improved perfusion of wound edges and a more physiological orientation of the incision—may contribute to reduced fascial retraction and potentially lower rates of late ventral hernia formation [17, 18, 49].

When DFC is not feasible, a strategy of planned ventral hernia remains an accepted alternative. Both the Eastern Association for the Surgery of Trauma (EAST) practice management guidelines and the WSES recommendations emphasize that early primary fascial closure should be pursued whenever possible; however, when closure cannot be achieved due to persistent visceral edema, contamination, or physiological instability, alternative strategies such as skin-only closure with subsequent ventral hernia repair are appropriate [2, 49].

Furthermore, this strategy is warranted upon the failure of dynamic traction techniques, evidenced by a lack of medial fascial progression despite repeated attempts, or in the presence of a high-output EAF that complicates the local wound environment and increases the risk of mesh contamination. In such scenarios, the focus shifts toward achieving secondary skin coverage over the granulated visceral mass—often via split-thickness skin grafts or secondary intention healing—postponing definitive abdominal wall reconstruction for 6 to 12 months until the inflammatory process has fully subsided [2, 49].

Ongoing research continues to explore potential biological and physiological adjuncts to improve OA outcomes. The Closed Or Open Laparotomy (COOL) trial, a large-scale, international multicenter randomized controlled trial, was initiated to investigate whether active NPWT in septic patients can facilitate the removal of inflammatory mediators from the abdominal cavity, thereby reducing the incidence of MOF. The study design compares the strategy of mandated open abdomen management with NPWT against the traditional approach of primary fascial closure in patients with severe peritonitis. While the trial has completed its recruitment phase in several centers, it remains in the final stages of data analysis and long-term follow-up; therefore, definitive results regarding its primary endpoints have not yet been fully published. If these hypotheses are confirmed, the findings may significantly influence future recommendations and potentially shift the current paradigm toward a more proactive OA strategy in selected septic populations [50].

Another emerging concept is direct peritoneal resuscitation (DPR), which involves instillation of a glucose-based dialysis solution into the open abdominal cavity to reduce local inflammation and intestinal edema. According to recent analyses, including those by Miguel Pera and colleagues, DPR may enhance visceral microcirculation, increase primary fascial closure rates, reduce ventilator days, and improve overall survival in emergency surgical patients [51]. Despite these promising physiological effects, strong clinical evidence remains limited. Current guidelines from the EAST do not recommend DPR as a standard intervention and instead emphasize mechanical fascial traction as the primary strategy to facilitate closure [49]. Ultimately, much of the existing evidence supporting DPR is derived from observational studies and retrospective registries, highlighting a significant lack of high-quality, multicenter randomized controlled trials required to validate its routine clinical application.

An additional innovative approach involves the use of Botulinum Toxin A (BTX) to reduce lateral abdominal wall tension. A systematic review by Simon Luton demonstrated that although maximal muscle paralysis develops gradually, a partial effect is already observable within 24 h. This early effect may facilitate fascial approximation during DCS where closure success rates with BTX have been reported to reach approximately 90.7%, compared with around 66% using standard techniques alone. Although the current evidence base remains limited to relatively small studies, BTX appears to be a safe and technically straightforward adjunct that may significantly enhance the success of abdominal wall closure when combined with NPWT [52].

Despite its physiological advantages in increasing intra-abdominal volume, vertical traction systems are associated with increased logistical complexity, higher procedural costs, and a more demanding nursing environment compared to traditional horizontal methods. Specifically, the logistical complexity arises from the requirement for specialized instrumentation and more frequent monitoring of system tension or pressure [28, 29]. From an economic perspective, procedural costs are significantly elevated due to the use of licensed proprietary technologies, whereas the established VAWCM technique utilizes readily available polypropylene mesh. Furthermore, the nursing burden is increased as patients treated with vertical systems often require specific positioning and intensive monitoring, placing a higher demand on ICU staff compared to the stable fascial fixation provided by the mesh-mediated approach. Consequently, long-term outcomes and direct comparisons between these newer systems and established gold standards like VAWCM remain critical areas for further clinical investigation.

### Algorithm for the Management of the Open Abdomen (OA)

The final subsection presents a proposed algorithm for OA management, synthesizing the key findings of this review into a structured, phase-based framework. As outlined in Table 3, the algorithm delineates the sequential stages of care—from initial decompression to definitive closure—highlighting principal indications, therapeutic objectives, and critical decision points that guide clinical management.

**Table 3:** Open Abdomen Management Algorithm. *Source: Own elaboration*

Phase	Title & Timeframe	Indications & Objectives	Key Actions & Decision Nodes
**I.**	**Decision & Initial Decompression**	IAH refractory to medical treatment, ACS, or Damage Control Surgery (DCS).	• Midline laparotomy, hematoma/effusion evacuation. • Source control. • TAC Initiation: NPWT with visceral protection (silicone layer).
**II.**	**Stabilization & Early Traction** (0–48 h)	Hemodynamic stabilization and correction of coagulopathy.	• Ready for traction? → YES: Initiate VAWCM (horizontal) or Fasciotens^®^ (vertical) during 1 st re-exploration. • **Ready?** → NO (Unstable/Ischemic): Continue standalone NPWT; re-evaluate in 24 h.
**III.**	**Serial Re-evaluations** (Every 48 h)	Washout and progressive medialization of fascial edges.	• Scheduled re-laparotomy. • Tightening the mesh or increasing vertical tension. • Nutritional Support: Early enteral nutrition (cautious approach).
**IV.**	**Definitive Closure** (Goal: Day 5–7 up to **Prolonged OA ≥ 28 days**)	Achieving primary fascial closure vs. salvage strategies.	• **Closure possible without tension?** → YES: Suture the native fascia (Primary Closure). • **NO (**Frozen Abdomen/Failed Traction**): Delayed fascial closure or Transition to **Planned Ventral Hernia** strategy (skin grafting or secondary intention).

*ACS* Abdominal Compartment Syndrome, *OA* Open Abdomen>*IAH* Intra-Abdominal Hypertension, *IAP* Intra-Abdominal Pressure, *ICU* Intensive Care Unit, *DCS* Damage Control Surgery, * DFC* Definitive fascial closure, *DPR* Direct peritoneal resuscitation, *TAC* Temporary Abdominal Closure, *TAR* transversus abdominis release, *CST* Component Separation Technique, *MOF*Multiple Organ Failure, *NPWT* Negative Pressure Wound Therapy, *VAWCM* Vacuum-Assisted Wound Closure and Mesh-Mediated Fascial Traction. The table outlines a simplified management algorithm for OA; individual clinical decisions should always be tailored to patient condition and institutional expertise.

## Conclusion

Management of the open abdomen represents a life-saving strategy in modern emergency surgery. Successful outcomes depend primarily on timely indication, meticulous critical care management, and the use of optimized temporary abdominal closure techniques. Current evidence supports the combination of NPWT with dynamic fascial traction as the most effective strategy for achieving delayed fascial closure. Achieving definitive fascial closure within the first postoperative week appears to significantly reduce the risk of severe complications, including EAF, and is associated with improved survival.

The introduction of large international registries, particularly those coordinated by the WSES has substantially improved the quality of available evidence and provided valuable insights into global clinical practice. However, it should be emphasized that the overall quality of available clinical studies in this field is often limited by methodological constraints. Consequently, registry data currently represents the highest level of available evidence for many aspects of OA management.

Nevertheless, significant challenges remain, including the heterogeneity of patient populations and regional differences in healthcare infrastructure, logistics, and surgical expertise. These factors highlight the ongoing need for further standardization of management protocols. Ultimately, high-quality, multicenter randomized controlled trials are urgently needed to further strengthen the evidence base and provide more definitive guidance for the management of these critically ill patients.

## Data Availability

No datasets were generated or analysed during the current study.
